# Influence of Different Tool Electrode Materials on Electrochemical Discharge Machining Performances

**DOI:** 10.3390/mi12091077

**Published:** 2021-09-07

**Authors:** Islam Md. Rashedul, Yan Zhang, Kebing Zhou, Guoqian Wang, Tianpeng Xi, Lei Ji

**Affiliations:** 1 School of Mechanical and Power Engineering, Nanjing Tech University, Nanjing 211800, China; rashed.eee38@gmail.com (I.M.R.); wangy_cgte@163.com (G.W.); xtp63@163.com (T.X.); jilei.1993@163.com (L.J.); 2 National Key Laboratory for Remanufacturing, Beijing 100072, China; zhoukb99@sina.com

**Keywords:** electrochemical discharge machining (ECDM), material removal rate (MRR), electrode wear ratio (EWR), overcut (OC), electrical properties, tool material

## Abstract

Electrochemical discharge machining (ECDM) is an emerging method for developing micro-channels in conductive or non-conductive materials. In order to machine the materials, it uses a combination of chemical and thermal energy. The tool electrode’s arrangement is crucial for channeling these energies from the tool electrode to the work material. As a consequence, tool electrode optimization and analysis are crucial for efficiently utilizing energies during ECDM and ensuring machining accuracy. The main motive of this study is to experimentally investigate the influence of different electrode materials, namely titanium alloy (TC4), stainless steel (SS304), brass, and copper–tungsten (CuW) alloys (W70Cu30, W80Cu20, W90Cu10), on electrodes’ electrical properties, and to select an appropriate electrode in the ECDM process. The material removal rate (MRR), electrode wear ratio (EWR), overcut (OC), and surface defects are the measurements considered. The electrical conductivity and thermal conductivity of electrodes have been identified as analytical issues for optimal machining efficiency. Moreover, electrical conductivity has been shown to influence the MRR, whereas thermal conductivity has a greater impact on the EWR, as characterized by TC4, SS304, brass, and W80Cu20 electrodes. After that, comparison experiments with three CuW electrodes (W70Cu30, W80Cu20, and W90Cu10) are carried out, with the W70Cu30 electrode appearing to be the best in terms of the ECDM process. After reviewing the research outcomes, it was determined that the W70Cu30 electrode fits best in the ECDM process, with a 70 μg/s MRR, 8.1% EWR, and 0.05 mm OC. Therefore, the W70Cu30 electrode is discovered to have the best operational efficiency and productivity with performance measures in ECDM out of the six electrodes.

## 1. Introduction

The manufacturing industry faces a challenge in creating holes or cuts in rigid materials with high geometrical precision. The process of electrochemical discharge machining (ECDM) is a combination of electrochemical and electro-discharge machining, with applications in aerospace, electronics, and miniaturized medical equipment, especially for drilling holes with complex shapes and geometries. However, due to the uncertain nature of the ECDM mechanism, electrode material selection is made on an empirical basis based on experimental results. As a result, it is critical to select an effective tool electrode to improve performances, such as the material removal rate (MRR), electrode wear ratio (EWR), overcut (OC), and surface defects on difficult-to-machine alloys.

According to the literature, the electrode content, electrode shape, and machining parameters all have a significant impact on electrical discharge machining (EDM) and ECDM efficiency. It can be inferred that there is a requirement for a comparative investigation about the effects of the tool material. In the hybrid EDM process, brass, copper (Cu), and copper–tungsten (CuW) are used [1]. For all current settings, the Cu electrode gave the highest MRR, followed by the brass and CuW electrodes, and CuW also had the lowest rate of EWR. The MRR and tools wear rate (TWR) was greatly influenced by the material properties of the electrode [2]. An inappropriate combination of the workpiece and the electrode leads to a reduced productivity in EDM sinking dies and excessive TWR. When executing the EDM operation with Ti-6Al-4V, using a cryogenically treated copper electrode produced an optimized MRR, improved surface quality, and reduced the EWR [3]. In the EDM performance of titanium grade six alloy, Cu anodes displayed a more moderate tool wear than brass and zinc anodes [4]. For both brass and zinc cathodes, a higher TWR was observed at a lower peak current (10 A). The magnitude of the TWR and overcut was of the order Cu > CuW > W, according to the experimental inquiry [5]. This occurred as a result of deviations in the thermal properties of the electrode materials. When drilling stainless steel with μ-EDM, the impact of the anode material and power discharge on the drilling time and TWR was investigated [6]. When a tungsten carbide electrode was used, the increased power discharge resulted in a significant reduction in drilling time, causing the TWR to be degraded and overlapped. The EDM of AISI 1040 stainless steel used an AlSiMg electrode prepared through the selective laser sintering (SLS) process, as well as a Cu and brass electrode [7]. The electrode that was the best EDM parameter setting found for the maximization of the MRR and minimization of the TWR, ROC, and Ra was a novel type of tool electrode in a rod made of carbon fibers [8]. The MRR shows that the investigated electrodes are comparable to tungsten (W), silver tungsten (AgW), and CuW electrodes in terms of process efficiency, and can be considered a possible complementary electrode material for EDM. An experiment in EDM was conducted in which a Nimonic C263 superalloy was machined with Cu, W, and CuW electrodes [9]. As a result of the electrode material and discharge current, the Cu electrode exhibited a higher MRR and a lower EWR. The effects of input variables, such as DC supply voltage, electrolyte concentration, pulse-on-time, pulse-off-time, and the inter-electrode gap using three different tool electrode (Cu, brass, W) materials, on developing the ECDM setup on the MRR were investigated, where the optimum MRR is depicted by a brass electrode through the KOH electrolyte and the proper set-up combination of voltage and pulse-on-time [10].

Regarding the machining of the Ti-6Al-4V titanium alloy, the optimal technological parameter combination in EDM with the AlCrNi-coated electrode was found to be the peak current (40 A), voltage (55 V), and pulse-on-time (1000 s). As a result of the electrical conductivity of the tool coating, the current was discovered to be the most important element in the EDM process with the coated tool [11]. A Taguchi L_18_ experimental design was used to compare the process performances of the EDM of HcHcr D2 steel (DIN EN ISO 4957) using different electrode materials (copper, graphite, and brass), dielectric fluids (distilled water and kerosene), peak current, and pulse-on-time, and the process performances were analyzed with respect to MRR, SR, and TWR. Graphite electrodes using distilled water as a dielectric fluid had a higher MRR of 0.0632 g/min and a lower SR of 1.68 μm and TWR of 0.012 g/min than brass and copper electrodes [12]. An investigation of the characterization of ternary metals (Cu–Ni–TiN) for EDM electrodes using powder metallurgy for the formation of Cu in Ni-TiN electrodes was conducted using a cold press at pressures of 18, 20, and 22 MPa, which led to the enhancement of mechanical properties, such as hardness, electrical properties, and other properties. The result show that the 80% Cu–3% Ni–17% TiN electrode at 18 MPa had the highest hardness (124.38 HV) and lowest electric resistivity (0.39188 cm), whereas the specimen with an increased Cu, with a ratio of 85% Cu–3% Ni–12% TiN at 20 MPa, had the highest density (8.5472 g/cm^3^) and lowest porosity (6.2922%) [13]. While the Nb powder was mixed in dielectric fluid, a composite layer of TiO_2_-TiC-NbO-NbC was coated on the Ti-64 alloy using two separate methods (electric discharge coating (EDC) and EDM processes). It has been discovered that, in the EDC process, the high peak current and high Nb powder concentration increase the material migration, resulting in the deposition of a crack-free thick layer (215 m) on the workpiece surface [14].

Because of the combined effects of the chemical and thermal energies of ECDM, its machining performance is faster and it has a lower processing cost, a simpler setup, and a higher MRR with low EWR than other methods, such as EDM and ECM, irrespective of the typical properties of newer materials. As a result, researchers have always been interested in the ECDM efficiency upgrade. Regarding the combined process of EDM ablation and ECM in an aerosol dielectric, the MRR was 3.7 times higher than that of EDM or ECM, the TWR was reduced by 53.3%, and the value of the corner arc radius decreased by 44.3% [15]. An experimental study comparing the effects of molybdenum and high carbon steel (HCS) electrode materials on the creation of micro-holes in glass by pulse ECDM was conducted, where molybdenum electrodes had minor tool corrosion compared to HCS electrodes, due to their higher melting point [16]. Four tool materials, namely copper, tungsten, stainless steel, and high carbon steel, were used to build a >300-μm-deep micro-hole in glass using gravity feed ECDM, where the tungsten and copper tools achieved the highest and lowest machining speeds of 95 μm/s and 20 μm/s [17]. Regarding the efficiency of gravity feed ECDM with three different tool materials, tungsten, tungsten carbide, and stainless steel, the tungsten carbide tool had the fastest machining speed because of its highest average current, and the stainless steel tool had the highest tool wear because of both its lowest melting point and its thermal conductivity [18]. Using a NaNO_3_ working fluid and an electrochemical discharge drilling system with a conductivity of about 4.0 mS/cm resulted in optimum quality holes in the nickel-based superalloy, with a low TWR of the brass electrode [19]. The effects of tool electrode electrochemical discharge machining process parameters, where the machining is performed through a chemical etching effect, were investigated, and it was discovered that the melting of the workpiece material due to electrical discharges and the influence of the ECDM process parameters are associated with the tool electrode [20]. For sound hole making and an improved electro-discharge machining efficiency, an optimal parameter setting has been established [21], and the impact of process parameters on various process performance characteristics, such as the material removal rate, surface roughness, surface crack density, white layer thickness, radial overcut, and hole taper, has been investigated.

According to published research, several attempts have been made to optimize EDM results by using different tool electrodes. In order to observe EDM results, the majority of researchers concentrate on machining parameter variation. Parameter variation is not the only crucial aspect for analyzing the machining performance; a proper electrode selection for different machining is also important. In this study, different material electrodes are proposed in the ECDM process, whereby the effects of the electrodes on the machining performance parameters, including the machining efficiency, machining accuracy, MRR, EWR, OC and surface behavior, are investigated. First, four different electrodes are compared on the command variable. However, it has been found that two of the very-low-conductive electrodes’ performances are very poor compared to other two. Secondly, an electrode that performs well is taken from the first step of the experiment and compared to the other two electrodes, where these three electrodes are made of the same alloy but have a different chemical composition.

The present article focuses on the experimental study: stainless steel, titanium, brass, and copper–tungsten alloy-made electrodes are used to confirm the electrical conductivity and thermal conductivity effect on the ECDM performance. Machining parameters, normal flushing pressure, dielectric fluid, and rotational speed have been kept at constant in order to analysis the ECDM performances. If the tool electrodes’ electrical conductivity is high, the local temperature rise would owe less to the faster heat dissipation to the workpiece. As a result, the MRR gain is higher with a lower EWR. On the other hand, if the thermal conductivity is low, the electrode’s body temperature would be raised rapidly due to the reduction in heat transfer to the workpiece. As a result, a lower MRR is associated with a higher EWR, resulting in an irregular surface with more cavities and cracks. Furthermore, a scanning electron microscopy (SEM) analysis of the drilled holes and electrodes is performed to determine micro-voids, cracks, and craters on the surface, as well as material migration on the tip of the electrodes. After analyzing the experimental findings, it was discovered that the W70Cu30 electrode performs the best, with a 70 μg/s MRR, 8.1% EWR, and 0.05 mm OC in the ECDM machining process.

## 2. Mechanism of Electrochemical Discharge Machining (ECDM)

**Figure 1:**
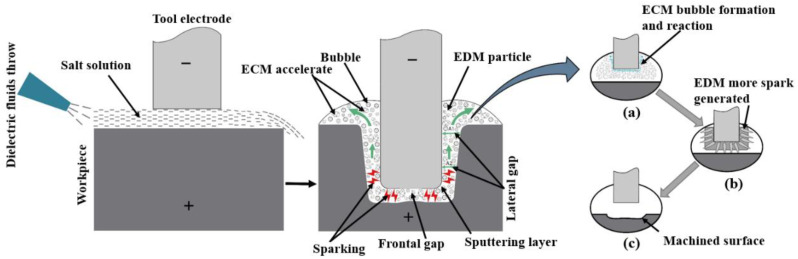
Schematic diagram of tool electrode high-speed electrochemical discharge machining mechanism. (**a**) ECM bubble formation and reaction, (**b**) EDM spark generating (**c**) Machined surface.

ECDM is a combined process of EDM and ECM, in which electrochemical dissolution and electrical discharge erosion occur simultaneously in ECDM in the lateral machining gap and frontal gap, respectively. The mechanism of ECDM is depicted in Figure 1. In this hybrid machining, sodium nitrate (NaNO_3_) has been used. NaNO_3_ is used for machining stainless steel when close replication of the tool is of utmost importance, as it prevents stray corrosion, ensuring precise tool replication. Lower concentration electrolytes are used to improve the machining resolution. The resolution is improved due to the increased electrolyte resistance, which requires shorter current paths for a given pulse length.

Both EDM and ECM are used to increase the lateral gap distance at the start of the drilling process. The lateral gap distance increases steadily with an increasing drilling depth due to the removal of materials from the electrochemical dissolution. When the lateral gap distance exceeds the theoretical maximum value, EDM becomes slow and eventually stops. Electrochemical dissolution will be the only process left in the side gap, and will start to remove the side wall material and expand the gap width. As a result, the conductivity of the solution, which affects electrochemical dissolution, is critical for minimizing electrode erosion and maintaining a good surface quality.

Since the electrode tip feeds towards the workpiece during the operation, the gap between the tool and workpiece is always kept at a gap distance smaller than the theoretical maximum value. This causes a spark discharge that is concentrated in the front gap. Electrolytic corrosion predominates in material removal, whereas the effects of electrochemical dissolution are relatively minor. In this process, bubbles are created by hydrogen evolution from an electrochemical reaction. The working fluid is constantly washed off from the process products, ensuring continuous spark discharge. Thus, the predominant material removal effect of electrostatic discharge erosion leads to a high drilling speed of the ECDM process.

In this ECDM process, the below electrochemical reaction equations are as follows:

At the anode:

The metal material can be dissolved as

Fe → Fe^2+^ + 2e^−^

Subsequently, the metal ions combine with the hydroxyl ions to precipitate as iron hydroxide,

Fe^2+^ + 2OH^−^ → Fe(OH)_2_↓

Hydroxyl ions evolve oxygen at the anode as

4OH^−^ → O_2_↑ + 2H_2_O + e^−^

At the cathode:

The reaction is likely to be the generation of hydrogen gas and hydroxyl ions.

2H_2_O + 2e^−^ → 2OH^−^ + H_2_↑

Following that, an electrochemical reaction could produce many bubbles, such as H_2_ and O_2_, surrounding the end tool electrode (anode). In the left part of Figure 1a, the formation of the bubble by the chemical reaction is depicted schematically where formation process of discharge channel are completed in the gas medium. The electrochemical discharges start small on the active electrode zone, and then more of the spark spreads out to a wide area of the workpiece (Figure 1b). This is due to the electrolyte’s lower electrical conductivity and material is removed from these discharges through melting, vaporization, and high-temperature chemical etching.

## 3. Experimental Setup

### 3.1. Machine Tool

**Figure 2:**
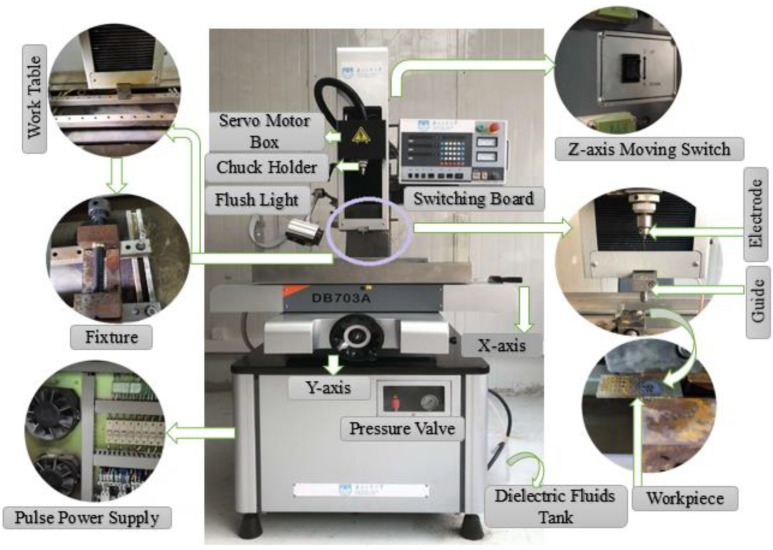
Photograph of the electrochemical discharge machining (ECDM) machine tool.

A machine tool has been shown in Figure 2, which includes an XYZ motion table, a working fluid recycling system, tool electrode clamp, high-pressure flushing unit, a power supply cell, voltage and current measuring unit, a pressure pump with pressure regulating valve, and a self-designed fixture. Tool electrode can be fixed into the machine head and can be carried in the feed rate guidance via a servomotor in the direction perpendicular to the XY plane. A motor is mounted on the *Z*-axis and rotates the tool electrodes. The control box contains the pulse generator, which provides the pulse voltage and the process control system. Due to the salt solution, all of the equipment’s exterior enclosures, especially the parts in contact with the solution, are constructed with a stainless steel portion.

### 3.2. Materials and Properties

**Figure 3:**
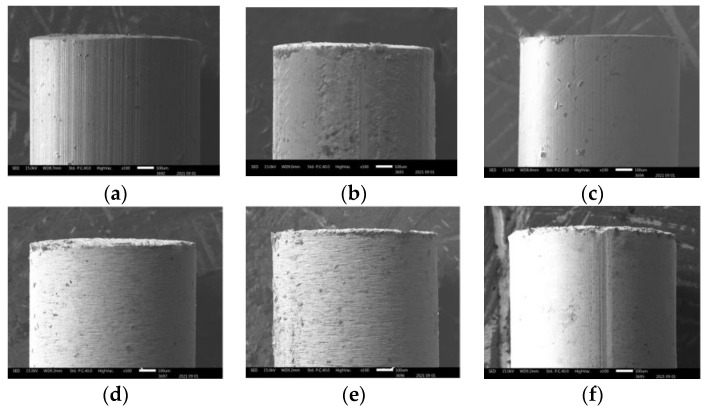
Scanning electron microscopy (SEM) picture of six different electrodes before machining: (**a**) SS304; (**b**) TC4; (**c**) brass; (**d**) W90Cu10; (**e**) W80Cu20; (**f**) W70Cu30.

**Figure 4:**
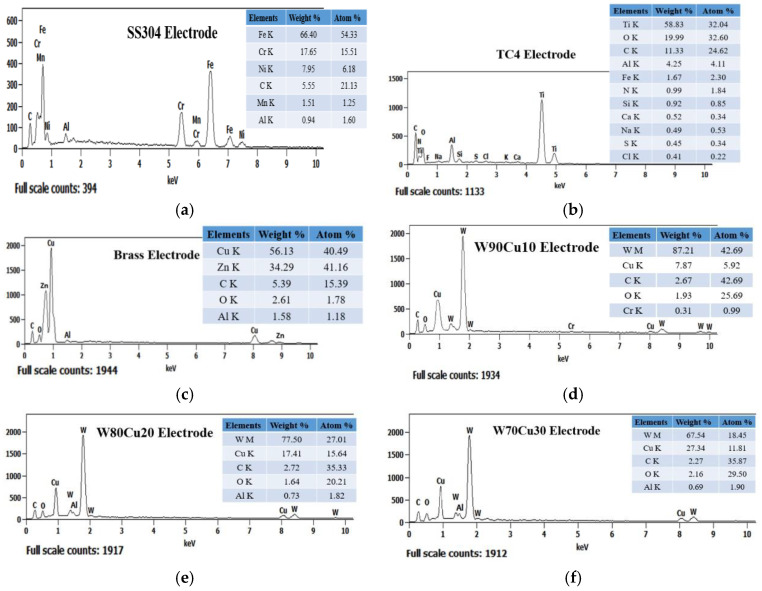
Energy-dispersive X-ray spectroscopy (EDX) spectrum analysis of six electrodes before ECDM: (**a**) SS304; (**b**) TC4; (**c**) brass; (**d**) W90Cu10; (**e**) W80Cu20; (**f**) W70Cu30.

All experiments were carried out on stainless steel (SS304) workpiece with the size of 43 mm (length) × 15 mm (width) × 2 mm (thickness), and all cylindrical electrodes were 1.0 mm in diameter and 200 mm in length. Figure 3 represents the six different tool electrodes before machining. SS304 has a high initial melting temperature, heat corrosion resistance, and strong properties, such as creep resistance and fatigue resistance (Figure 3a). The TC4 has excellent corrosion resistance, and can withstand high temperature (Figure 3b). Brass is copper alloyed with high Zinc content, which is most widely regarded for non-traditional machining with various electrodes (Figure 3c). Compared with copper and other traditional electrode materials, CuW alloy has a maximum melting point, and is a relatively cost-effective and efficient choice as electrode material (Figure 3d–f). As shown in Figure 4, EDX analysis of six types of electrodes was performed to investigate the accurate mass percentage of element contents before ECDM. Moreover, from the EDX spectrum, how much the other particle added to the tip part of the electrode after machining was confirmed. Electrode materials’ properties have been given in Table 1.

### 3.3. Measurements and Acquisitions

The main machining performance of the ECDM process was assessed by the MRR, EWR, overcut, surface defects, and machining quality of holes. The assessment of MRR and EWR was conducted on weight basis using high precision weight balance. Every electrode and workpiece was measured before and after machining carefully. After one single drilling, it is difficult to obtain weight of electrode and workpiece, which is why four repetitions were carried out by one electrode every time. Then, the electrode and workpiece weights were measured. However, the machining time was counted with every single drilling finish and calculated by mean statistical method. After that, the following equations were used to find out MRR, EWR, and OC.

The following equation gives the material removal rate of the workpiece:

MRR = (W_i_ − W_f_) ÷ T (1)

Here, W_i_ is the initial weight of the workpiece, W_f_ is the final weight of the workpiece after machining, and T is machining time.

Electrode wear ratio is given by the following equation:

EWR = {(E_wb_ − E_wa_) ÷ (W_i_ − W_f_)} × 100 (2)

Here, E_wb_ is the electrode weight before machining and E_wa_ is electrode weight after machining.

Overcut (OC) is calculated using the following equation:

OC = (D_w_ − D_t_) ÷ 2 (3)

Here, D_w_ is the frontal hole diameter of the workpiece and D_t_ is the tool electrode diameter. Overcut per side has been specified.

The tool electrode material electrical conductivity value is calculated using the below equation:

S = L ÷ (R × A)(4)

Here, L and A are the length and diameter of tool electrode, S is the electrical conductivity, and R is the electrical resistance.

### 3.4. Machining Procedure and Condition

The material removal mechanism of ECDM, surface defects in workpiece and tools, and machining efficiency were investigated. The machining process is carried out in two stages. First of all, copper, titanium, stainless steel, and tungsten alloys were commonly used as electrode materials; thus, the brass, TC4, SS304, and W80Cu20 are compared in order to obtain the better electrode material choice. Further, for the tungsten alloys, the W70Cu30, W80Cu20, and W90Cu10 are investigated as the electrode materials, according to the MRR, EWR, and OC, whereby the optimal electrode is identified. The workpiece and electrodes were observed by a scanning electron microscope (SEM), model JSM-IT300, JEOL, Japan and the material composition of the tool tips after machining was analyzed by energy-dispersive X-ray spectroscopy (EDX) system integrated with this SEM. The conductivities of salt solution were measured using the conductivity test instrument SevenCompact S230, produced by Mettler Toledo Company. A series of experiments were carried out to evaluate the performance of different electrode materials using the machining conditions listed in Table 2.

## 4. Result and Discussion

### 4.1. Comparison of MRR, EWR, and OC by W80Cu20, Brass, TC4, and SS304 Electrodes

**Figure 5:**
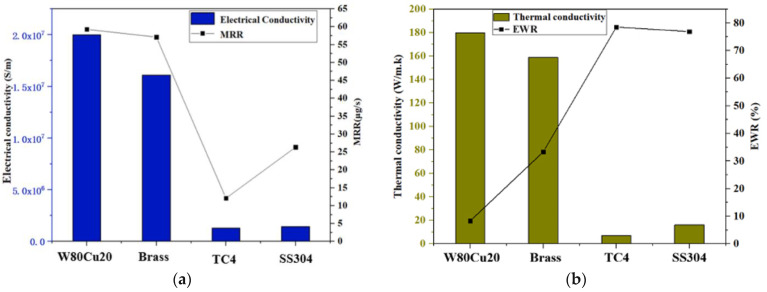
Influence of electrical conductivity and thermal conductivity of different electrodes under machining conditions Ton = 12 μs, Toff = 12 μs, Ip = 14.17 A; (**a**) MRR and (**b**) EWR.

In the ECDM process, the effect of the tool materials’ electrical conductivity and thermal conductivity on the MRR and EWR for W80Cu20, brass, TC4, and SS304 are shown in Figure 5. In Figure 5a, the MRR decreases as conductivity decreases, recording at 59.3, 57.4, and 12.1 μg/s for W80Cu20, brass, and TC4 electrodes, respectively. Following this, the MRR increases up to about 26.35 μg/s due to the higher conductivity of the SS304 electrode over the TC4 electrode. The higher the electrical conductivity of the electrode material, the faster the thermal energy is dissipated to the machining zone. This thermal energy evaporates the water from the electrolyte at the machining zone. Thus, only molten salt (sludge) is left at the tool–work material interface. The accumulation of this sludge ceases bubble formation beneath the tool electrode. Hereafter, bubbles evolve only from the edges and side walls of the tool electrode. These bubble coalesce and form thicker films and initiate more discharges at the tool edges. Hence, the amount of thermal energy reaching the workpiece material is cut down, and the majority of this energy is dissipated in the electrolyte by convection. W80Cu20 transfers more heat energy to the workpiece during the machining process. As a result, W80Cu20 generates a higher MRR by melting the workpiece.

The high thermal conductivity of the electrode materials is crucial in determining the EWR. The TC4 electrode has the highest EWR (78.54%), followed by SS304 (76.92%), brass (33.33%), and W80Cu20 (8.33%), as shown in Figure 5b. The tool electrode’s thermal conductivity determines the electron’s drifting ability in any substance. Due to the tool electrodes’ low thermal conductivity, TC4 and SS304 electrodes represent the highest EWR. These two electrodes’ drifting electrons are very low in bulk material for discharging, and the temperature increases towards the electrode’s own body. Following that, the TC4 and SS304 electrodes burn during the machining mode with a lower MRR and a higher electrode wear ratio. The brass electrodes’ high thermal conductivity allows for more heat to be applied to the workpiece, resulting in faster machining and a far lower EWR. However, W80Cu20 has the highest thermal conductivity (180 Wm^−1^k^−1^) compared to the other three electrodes, followed by brass (159 Wm^−1^k^−1^), SS304 (16.2 Wm^−1^k^−1^), and TC4 (7 Wm^−1^k^−1^), respectively. Heat cannot pass through a material with a low thermal conductivity without damaging the electrode. The low EWR is due to the higher thermal conductivity of W80Cu20.

**Figure 6:**
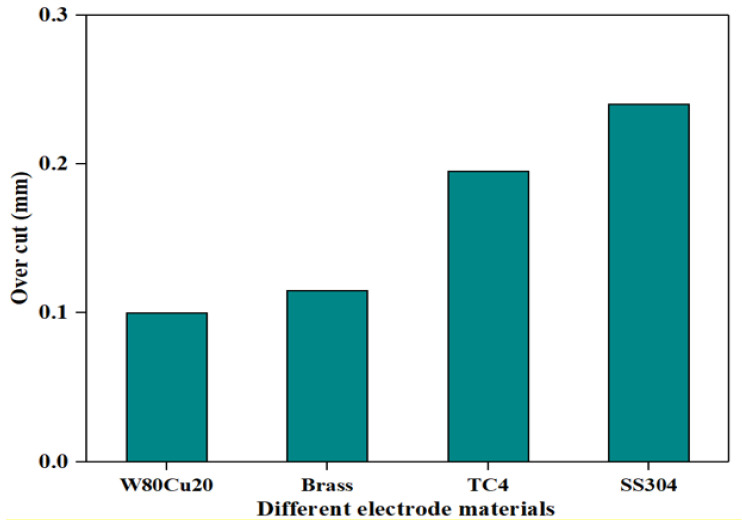
Different tool electrodes’ effects on side overcut under machining conditions T_on_ = 12 μs, T_off_ = 12 μs, I_p_ = 14.17 A.

**Figure 7:**
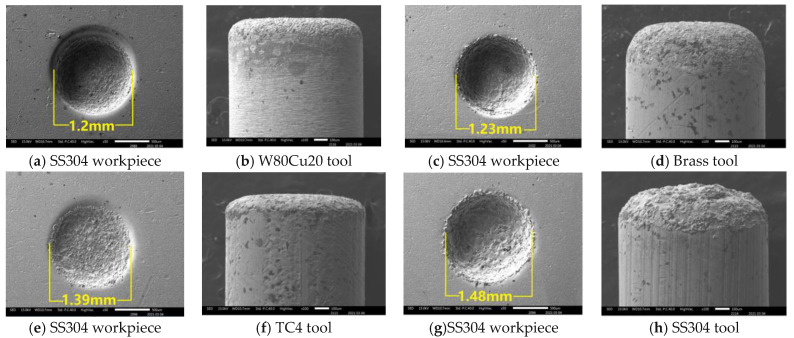
SEM picture of different electrodes and discharging phenomena of the holes on SS304 workpiece under machining conditions Ton = 12 μs, Toff = 12 μs, Ip = 14.17 A.

In the ECDM process, the overcut is an essential factor that affects geometrical accuracy. By comparing the overcut caused by the four electrodes, it can be seen in Figure 6 that the SS304, TC4, and brass electrodes have created relatively larger overcuts (0.240, 0.195, 0.115 mm), whereas the W80Cu20 electrode produces a lower overcut (0.100 mm). Figure 7c,e,g show that the brass, TC4, and SS304 electrodes have made large diameters of 1.23, 1.39, and 1.48 mm, respectively, whereas the W80Cu20 (Figure 7a) electrode has created a 1.20 mm diameter on the workpiece. For W80Cu20, the heat from the electrochemical discharges is quickly removed from the machining zone, but for the other three electrodes, the electrochemical discharges are slowly removed. As a result, electrode and hole wall side sparking of the workpiece occurs more often for the SS304, TC4 and brass electrodes than the W80Cu20 (Figure 7a) electrode. The hole wall surface roughness, side gap spark, and electrode erosion are higher for brass, TC4 and SS304 electrode as shown in Figure 7c,e,g. The thermal conductivity of the SS304, TC4, and brass electrode materials are low, as well as the number of the drifting electrons, resulting in a lower discharging in the tip than in the W80Cu20 electrode. The corner of the electrode end has the highest current density and suffers the maximum wear. From the observations in Figure 7d,f,h, the brass, TC4, and SS304 electrodes’ tip part eroded with more roughness than that of the W80Cu20 (Figure 7b) electrode. W80Cu20 has the largest thermal conductivity and its melting point is higher than the other three electrodes.

It is concluded that it is essential for the material that acquires good electrode wear properties in a machine with a high electrical conductivity to be complex. The W80Cu20 acquires a higher electrical conductivity (2.0 × 10^7^ S/m) than the brass (1.61 × 10^7^ S/m), SS304 (1.45 × 10^6^ S/m), and TC4 (1.28 × 10^6^ S/m) electrodes. A higher electrical conductivity enables a quick heat transfer through all of the workpiece. Subsequently, the lowest EWR with a higher MRR and minimum overcut are obtained by the W80Cu20 electrode.

### 4.2. Surface Defects on Workpiece and Material Transfer on Electrodes

**Figure 8:**
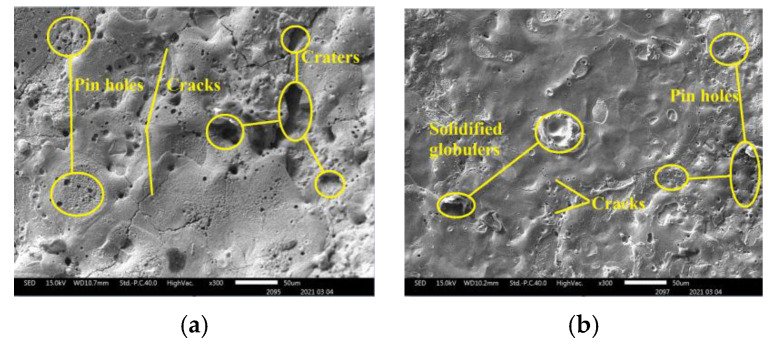

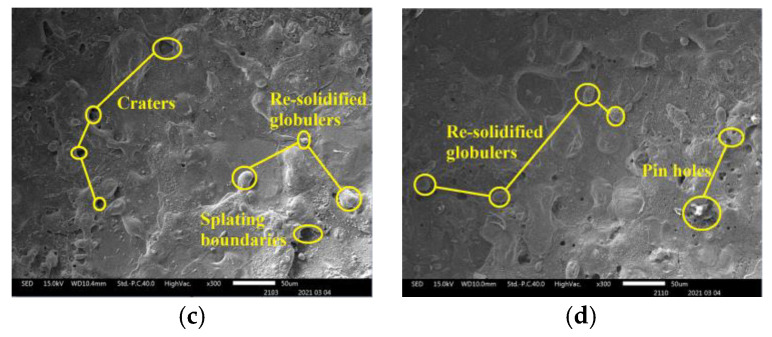
The surface of SS304 machined by different electrodes: (**a**) SS304; (**b**) TC4; (**c**) brass; (**d**) W80Cu20.

In Figure 8, SEM images are used to study the surface topography of the machined SS304 alloy. The electrode material properties have an impact on the surface roughness. If the electrode material’s electrical conductivity is higher, it allows for effective discharges while lowering ineffective pulses, resulting in an improved workpiece surface quality. The electrodes with the most cracks and pinholes on the machined surface are SS304 (Figure 8a) and TC4 (Figure 8b), followed by brass and W80Cu20 electrodes. The hydrodynamic regime is vigorously non-desirable. On the one hand, it increases the drilling time, and on the other hand, it is responsible for lowering the micro-aperture quality, as structural damage (micro-cracks and pinholes) of the drilled micro-apertures transpires in this regime. This phenomenon is caused by the formation of distinct thermally impaired areas. The erosion of the TC4 and SS304 electrodes is high with a lower MRR because the discharging effect is very weak with higher surface defects. However, machining with brass (Figure 8c) and W80Cu20 (Figure 8d) electrodes creates fewer craters and a smaller pinhole. The W80Cu20 electrode has a better surface than the other three electrodes. The W80Cu20 electrode provides the best surface with the least amount of erosion and the highest MRR.

**Figure 9:**
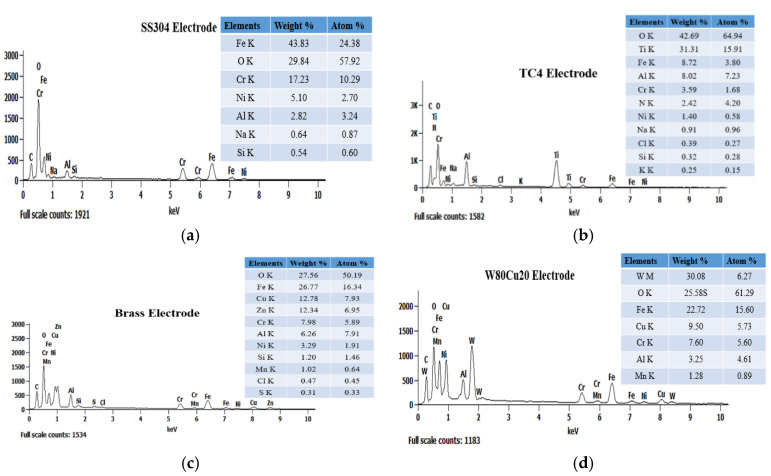
EDX spectrum analysis of four electrodes after ECDM: (**a**) SS304; (**b**) TC4; (**c**) brass; (**d**) W80Cu20.

In terms of surface composition, Figure 9 shows the EDX spectrum analysis results for four different electrodes used in the ECDM process. The unexpected oxygen accumulation on the electrode surface can be explained by the reformulation layer formation mechanism. During an electrical discharge, high temperatures in the discharge channel induce the melting and evaporation of the metal from the surface; at the same time, the electrolyte decomposes into oxygen and hydrogen at these high temperatures. The TC4 electrode produced the highest oxygen layer (42.69%) among the four electrodes, whereas SS304 produced the second highest oxygen layer (29.84%). The lowest oxygen element composition was achieved using brass and W80Cu20 electrodes (27.56 and 25.58%). Due to their low conductivity and worse machining instability, the tip part of the SS304 and TC4 electrodes over-melted, and other particles attached more. Comparing the brass and W80Cu20 electrode, the brass electrode has the lowest melting point and thermal conductivity; as a result, there are a large amount of other substances associated with the brass electrode.

The excellent electrical conductivity and thermal conductivity of the electrode materials facilitate uniform and effective discharges, minimizing short pulses and enhancing the workpiece surface nature. As a result, choosing a tool electrode that is close to the manufacturing or machining process is needed.

### 4.3. MRR, EWR, and OC Machining with W70Cu30, W80Cu20, and W90Cu10 Electrodes

**Figure 10:**
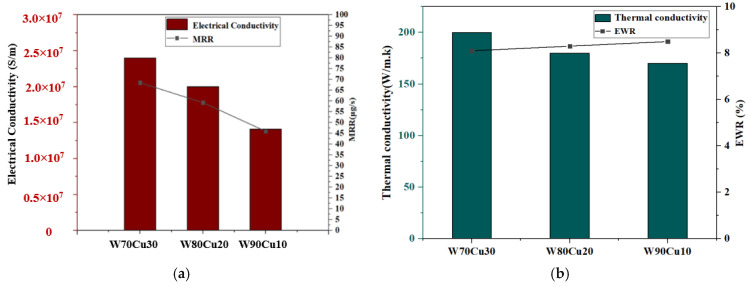
Influence of electrical conductivity and thermal conductivity under machining conditions T_on_ = 12 μs, T_off_ = 12 μs, I_p_ = 14.17 A; (**a**) MRR and (**b**) EWR for W70Cu30, W80Cu20, and W90Cu10 electrodes.

Since tungsten alloy is better than other alloys, W70Cu30, W80Cu20, and W90Cu10 made up of the same elements with different tungsten and copper contents were further investigated. Figure 10 shows the experimental results in order to explain the effects of electrode materials on the MRR and EWR. W70Cu30 has been found to be the most superior among the three electrodes, due to its high electrical conductivity (Figure 10a), and the material removal rate by the W70Cu30 electrode is about 70 μg/s, which is higher than that of the W80Cu20 (59.3 μg/s) and W90Cu10 (45 μg/s). W70Cu30′s high conductivity allows for rapid electron flow and heat dissipation on the workpiece. The amount of electric energy lost as electrothermal energy at the tool electrode (W70Cu30) is lower; thus, most of the energy is dissipated on the bulk of the workpiece for eroding the material.

The W90Cu10 and W80Cu20 electrodes’ EWR is higher than W70Cu30, as shown in Figure 10b, with 8.5, 8.3, and 8.1%, respectively. Thermal conductivity is inversely proportional with electrical resistivity, but proportional with electrical conductivity. W70Cu30′s low resistivity allows for rapid heat dissipation to the workpiece material, lowering the EWR and improving the tool shape retention. When the tool electrode material’s thermal conductivity is high, the electron flow through the material is high; thus, the majority of the heat has dissipated by an electron hitting the workpiece, which is used to melt the particle.

**Figure 11:**
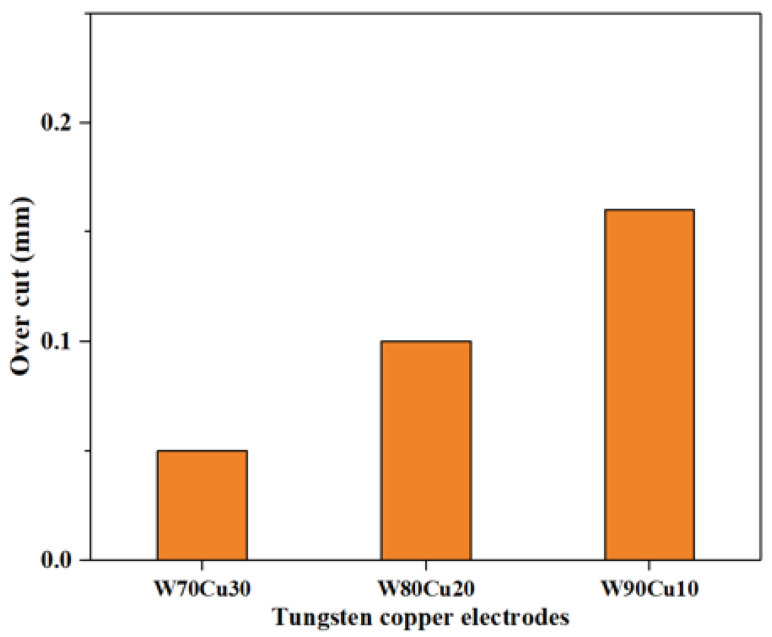
Tungsten–copper tool electrodes’ effect on side overcut under machining conditions T_on_ = 12 μs, T_off_ = 12 μs, I_p_ = 14.17 A.

**Figure 12:**
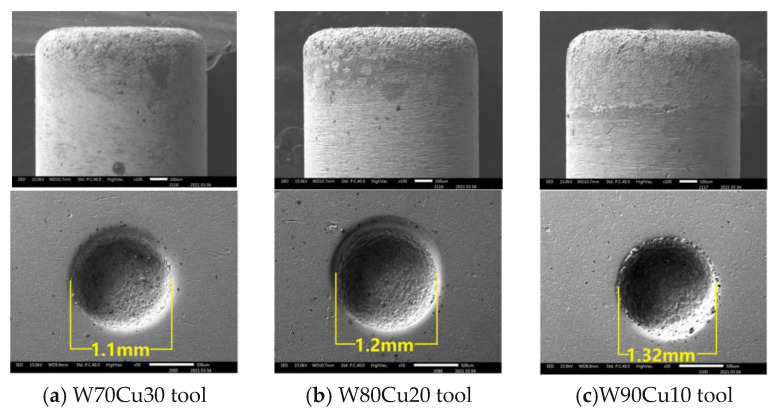
SEM images of three types of CuW electrodes and discharging phenomena of the hole on SS304 workpiece under machining conditions T_on_ = 12 μs, T_off_ = 12 μs, I_p_ = 14.17 A.

Concerning the geometrical characteristics of holes, the manufacturing process is more accurate when the CuW electrode is used for the overcut (Figure 11), and diameters are lower (Figure 12). From the SEM images (Figure 12) of three CuW electrodes and the machining workpiece hole, the W70Cu30 electrode machining hole diameter (Figure 12a) is 1.1mm, which is lower than the other two holes’ diameter, i.e., 1.2 and 1.32 mm (Figure 12b,c), respectively. This occurs on the machining instant, because the edge sparking for W80Cu20 and W90Cu10 appeared more than W70Cu30. The machining time for W80Cu20 and W90Cu10 is also more than W70Cu30. It can be seen from the SEM images of the three-electrode tip and upper body part that the W90Cu10 and W80Cu20 electrodes’ surfaces are rougher than that of W70Cu30. Due to large diameter holes processing by W90Cu10 and W80Cu20, the overcut is also higher for these two electrodes (0.1 and 0.16 mm) than W70Cu30 (0.05 mm), as shown in Figure 11. A high thermal diffusivity means that heat moves rapidly through the electrode without damaging it. A lower thermal diffusivity of W80Cu90 and W80Cu20 results in the two electrodes’ side sparking between the hole wall and electrode happening more frequently than the tip part, when compared to W70Cu30. Hence, the OC is lower for W70Cu30 than the other two electrodes. When concerning machining complex geometrical materials, choosing suitable electrodes is extremely important. The main reason is that, due to the higher conductivity of W70Cu30, the corner end of the electrode is not more eroded.

It is concluded that the tool’s thermal conductivity regulates the amounts of thermal energy extracted from the electrochemical discharge, as well as the temperature variations in the workpiece in front of it, resulting in viscosity changes. As a result, it is likely that the tool’s heat conductivity will influence how the drilling progresses. The W70Cu30 acquires a higher thermal conductivity (200 W/m·k.) than the W80Cu20 (180 W/m·k.) and W90Cu10 (170 W/m·k.) electrodes. Subsequently, the lowest EWR is obtained by the W70Cu30 electrode.

### 4.4. Surface Defects on Workpiece and Material Transfer on W90Cu10, W80Cu20, and W70Cu30 Electrodes

**Figure 13:**
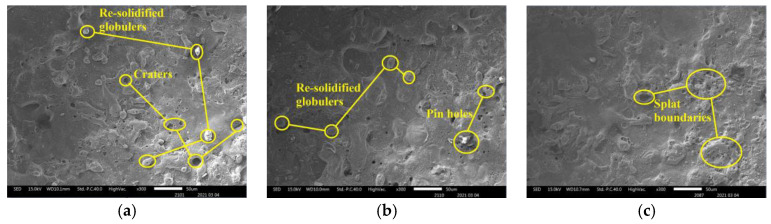
The surface of SS304 alloy machined by different electrodes: (**a**) W90Cu10; (**b**) W80Cu20; (**c**) W70Cu30.

SEM and EDX are used to examine the surface topography of machined SS304 alloy workpiece, as seen in Figure 13. The surface produced by various electrode machining on the workpiece with the static fluid machining was covered by pinholes, craters, and a re-solidified layer containing oxidized and hydroxides generated by the ECDM process. W90Cu10 (Figure 13a) and W80Cu20 (Figure 13b) electrodes have contributed to generate more craters and pinholes on the machined surface. As a result of improper thermal energy distribution and the shortage of electrolytes inside the micro-hole during drilling, a limited amount of materials were removed by chemical etching and a generous amount of surface deformities occurred. Furthermore, under the high temperature and rapid cooling process, the unsatisfactory flushing condition affects the removal of the machining by-products, and sections of the machining by-products cohere on the surface. The discharging effect is very poor with higher surface defects, and the W90Cu10 and W80Cu20 electrodes’ erosion is high with a lower MRR. The W70Cu30 electrode produces the optimal surface, with the most negligible erosion and the maximum MRR.

**Figure 14:**
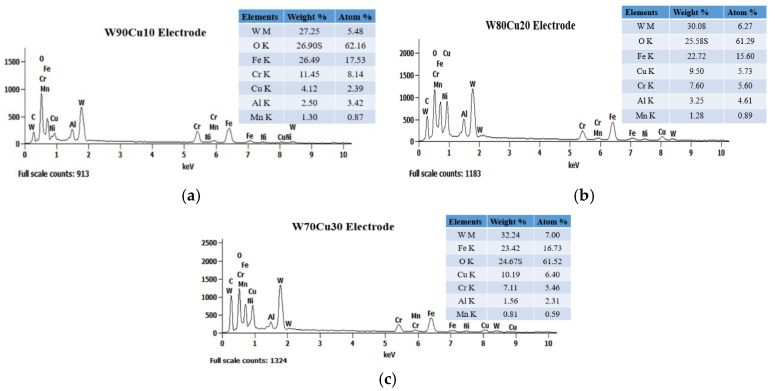
EDX spectrum and material deposition analysis of three electrodes after ECDM process: (**a**) W90Cu10; (**b**) W80Cu20; (**c**) W70Cu30.

Figure 14 shows the EDX spectrum analysis results for three different electrodes. The reformulation layer formation mechanism can explain the unexpected oxygen deposition on the electrode surface. High temperatures in the discharge channel cause the melting and evaporation of the metal from the surface during electrical discharge; simultaneously, the electrolyte liquid decomposes into oxygen and hydrogen at these elevated temperatures. Between all of the tungsten–copper electrodes, the W70Cu30 electrode generates the lowest oxygen layer (24.67%), followed by W80Cu20 (25.58%) and W90Cu10 (26.90%). On the other hand, the W90Cu10, W80Cu20, and W70Cu30 electrodes have the most significant effect on the manganese metal, with W70Cu30 having the least (0.81%) connection. The W70Cu10 electrode has a lower (1.36%) aluminum attachment than the other two electrodes. There was also a lower chromium migration to W70Cu30. Among the three materials studied, W80Cu20 and W90Cu10 have the lowest thermal conductivity and machining instability, and also a higher EWR than W70Cu30. During the ECDM process, the tip part of the two electrodes (W80Cu20 and W90Cu10) had melted more, and other machining zone’s particles attached more easily than W70Cu30. As a result, W70Cu30 is the most effective electrode for ECDM.

It is concluded that the excellent electrical and thermal conductivity of the electrode material promote uniform and effective discharges, enhancing the machined surfaces’ nature. Therefore, selecting a tool electrode that is close to the manufacturing or machining process is needed. W70Cu30 is the best electrode, and represents the optimal result in ECDM performances.

## 5. Conclusions

The selection of the appropriate electrode material is crucial in determining the ECDM performance. In this study, selecting a suitable electrode for the ECDM process has been proposed. Several experimental investigations have been conducted on the corresponding workpiece by the influencing of six different electrode materials, namely TC4, SS304, brass, W80Cu20, W70Cu30, and W90Cu10. Finally, W70Cu30 seems to be well-suited for ECDM, in that it not only achieves a good MRR and suffers the lowest EWR, but also shows the best machining stability. The experimental analysis supports the following inference:

(1) As a result of higher conductivity, the discharge channel forms quickly as the electrical conductivity increases, the discharge delay time decreases, and the discharge energy emitted to the workpiece at the same time increases, resulting in an increase in the MRR. Here, the highest MRR of about 70 μg/s is obtained when using the W70Cu30 electrode over using the other five electrodes;
(2) All CuW electrodes exhibit the lowest EWR, followed by the brass, TC4, and SS304 electrodes. However, among all CuW electrodes, W70Cu30 has shown a lower EWR (8.1%) than the other two electrodes because this electrode has a very high thermal conductivity. Due to its high thermal conductivity, the heat produced during machining diffuses into the space, decomposing the electrolyte fluids’ oxygen at a very high temperature, with some accumulating around the electrode, preventing further electrode erosion;
(3) All CuW electrodes represent the lowest side overcut, followed by the other three electrodes (brass, TC4, and SS304). Among all CuW, W70Cu30 exhibits the least overcut (0.05 mm). W70Cu30 has miniature craters with no cracks and a less rough surface. The unexpected oxygen deposition is the lowest for W70Cu30, followed by the other electrodes. Other material compositions to this electrode are in minimal percentages.

## Figures and Tables

**Table 1:** Properties of the electrode materials.

Properties	TC4	SS304	Brass	W70Cu30	W80Cu20	W90Cu10
Density (g/cm^3^)	4.43	8	8.73	13.80	15.15	16.75
Melting point (°C)	1660	1455	904	3420	3420	3420
Thermal conductivity (W/m K)	7	16.2	159	200	180	170
Specific heat capacity (J/kg K)	553	500	920	232	190	155
Tensile strength (Mpa)	862	515	360	516	620	700

**Table 2:** Machining parameters for electrochemical discharge machining (ECDM) experiments for brass, TC4, SS304, W70Cu30, W80Cu20, and W90Cu10 electrodes.

Machining Parameters	Fixed Parameters
Pulse width, T_on_ (μs)	12
Pulse interval, T_off_ (μs)	12
Peak current, I_p_(Amp.)	14.17
Open circuit voltage, V_OC_(V)	85
Electrolyte, NaNO_3_ (g/L)	4
Regulation per minute(RPM)	300

## Data Availability

The data presented in this study are available in this published article.
